# Supplementary material for: Dual-targeting snRNA gene therapy rescues STMN2 and UNC13A splicing in TDP-43 proteinopathies
Source: bioRxiv. 2025 Dec 3:2025.12.01.691001. Preprint. [Version 1] doi: 10.64898/2025.12.01.691001 (PMC12822690; doi:10.64898/2025.12.01.691001)
Supplement: Supplementary file 1 [file NIHPP2025.12.01.691001v1-supplement-1.pdf]

bioRxiv preprint doi: https://doi.org/10.64898/2025.12.01.691001; this version posted December 3, 2025. The copyright holder for this preprint (which was not certified by peer review) is the author/funder, who has granted bioRxiv a license to display the preprint in perpetuity. It is made available under a CC-BY 4.0 International license.

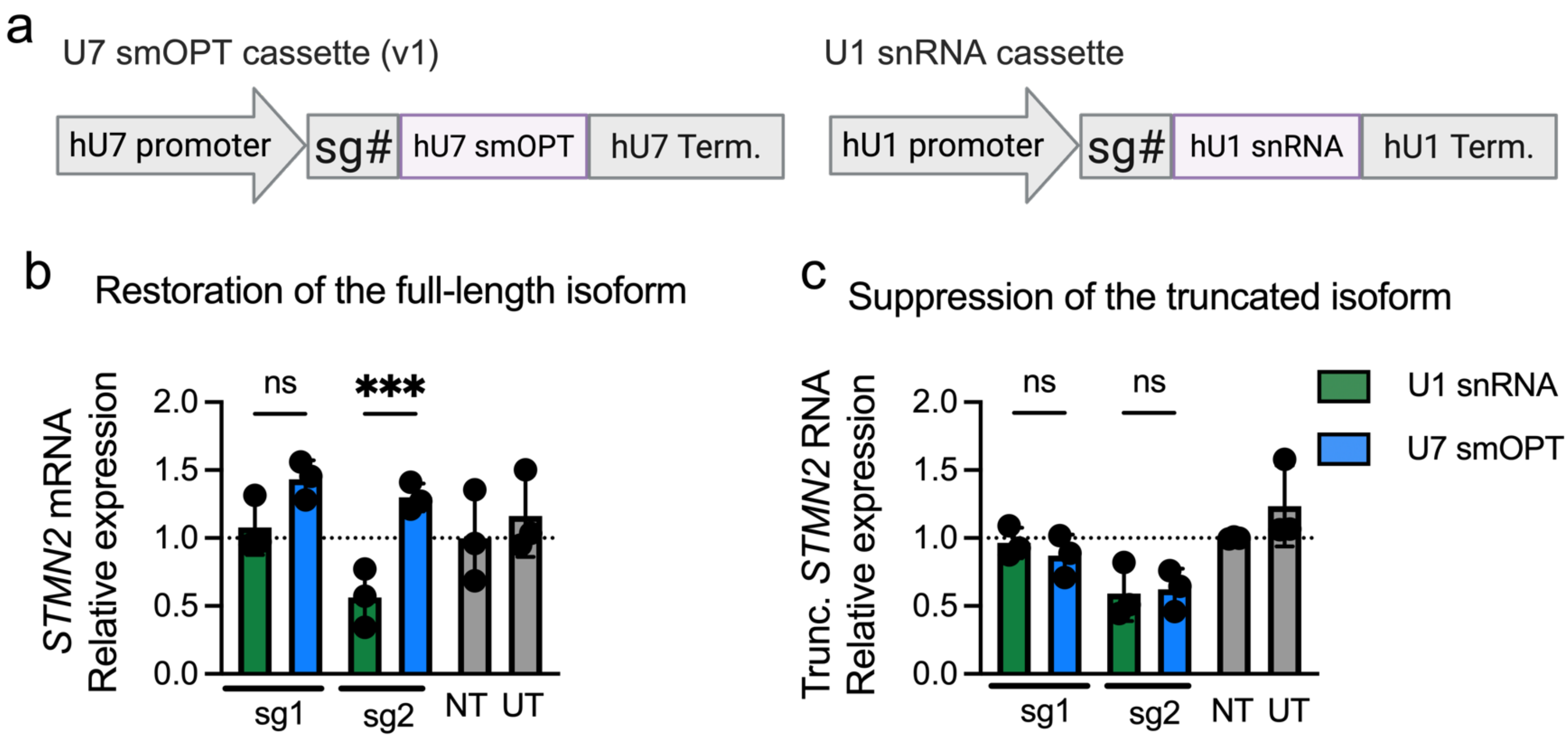

Figure S1: U1 snRNAs suppress cryptic exon without increasing full-length STMN2.
a) Schematic of U7smOPT and U1 snRNA expression cassettes.

b,c) Bar plots showing levels of (b) the full-length *STMN2* isoform or (c) the truncated *STMN2* isoform in SH-SY5Y$^{N352S/N352S}$ cells after treatment with lentivirus expressing U7smOPT or U1 snRNAs targeting exon 2a. The NT snRNA is an U1 snRNA with a 15 base pair non-targeting sequence. N=3 biological replicates. One-way ANOVA with Sidak correction. ***P < 0.001; ns, not significant.

bioRxiv preprint doi: https://doi.org/10.64898/2025.12.01.691001; this version posted December 3, 2025. The copyright holder for this preprint (which was not certified by peer review) is the author/funder, who has granted bioRxiv a license to display the preprint in perpetuity. It is made available under a CC-BY 4.0 International license.

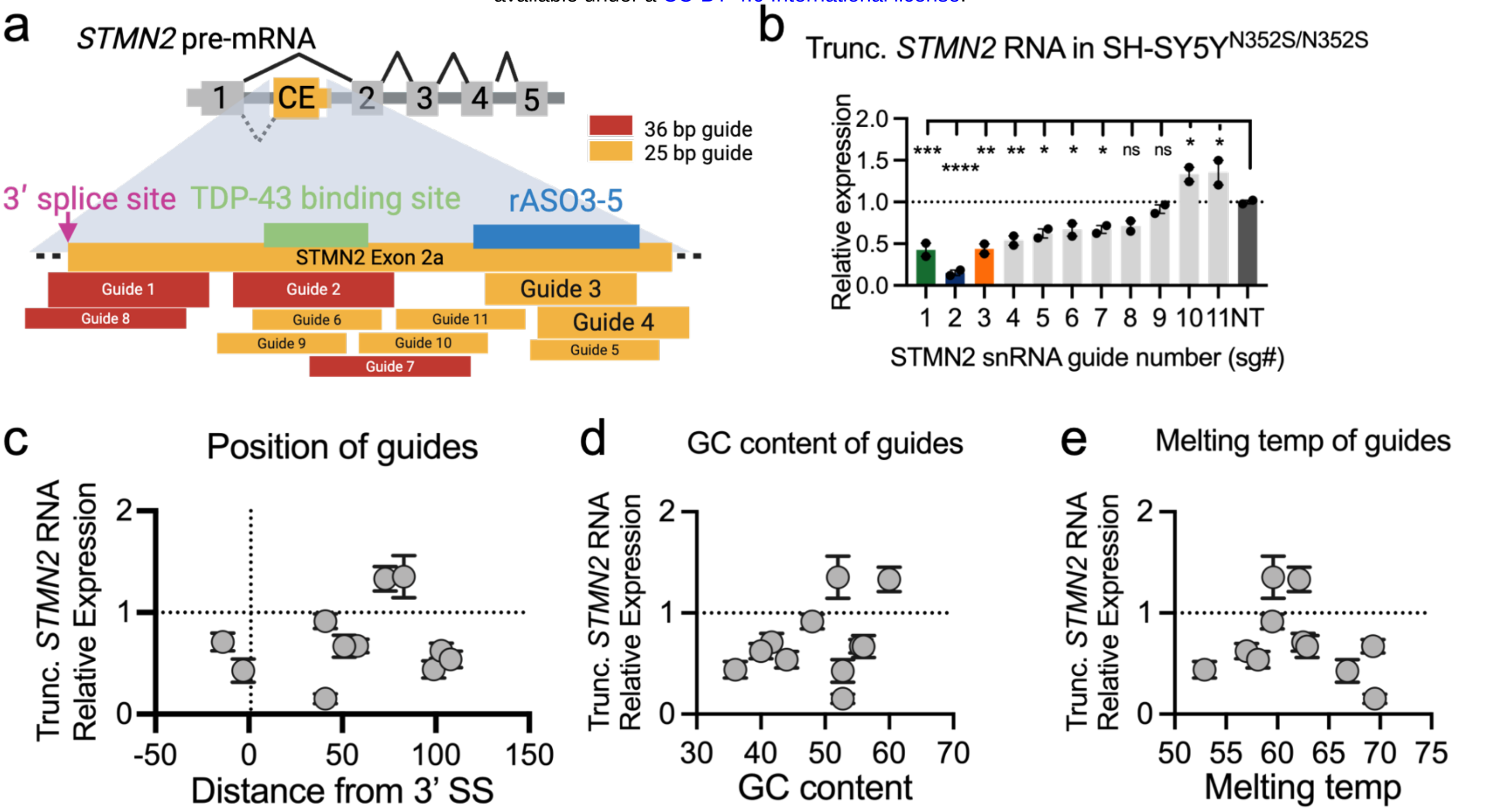

Fig S2: snRNAs of various qualities suppress STMN2 cryptic splicing.

a) Schematic representation of the *STMN2* cryptic exon and the location of 10 snRNA targets. TDP-43 binding sites and the targeting site of rASO3-5 from Baughn et al., 2023 are indicated. Guides of 25 (orange) or 36 base pairs (red) were tiled along the CE region.

b) Bar plot showing levels of the truncated *STMN2* isoform in SH-SY5Y$^{N352S/N352S}$ cells transduced with U7smOPT expressing lentivirus targeting the *STMN2* cryptic exon region. Data are rt-qPCR of truncated *STMN2* mRNA normalized to GAPDH (ΔC_q), relative to normalized levels in cells transduced with non-targeting (NT) smOPT ($\Delta\Delta C_q$). N=2 biological replicates. One-way ANOVA with Dunnett's correction. ****$P < 0.0001$; ***$P < 0.001$; **$P < 0.01$; *$P < 0.05$; ns, not significant.

c-e) Scatter plots of levels of the truncated *STMN2* isoform in SH-SY5Y$^{N352S/N352S}$ cells following lentiviral delivery of the 10 snRNA guides, based on their (c) distance from the 3' splice site, (d) GC content, and (e) predicted melting temperature. Each point represents N=2 biological replicates.

bioRxiv preprint doi: https://doi.org/10.64898/2025.12.01.691001; this version posted December 3, 2025. The copyright holder for this preprint (which was not certified by peer review) is the author/funder, who has granted bioRxiv a license to display the preprint in perpetuity. It is made available under a CC-BY 4.0 International license.

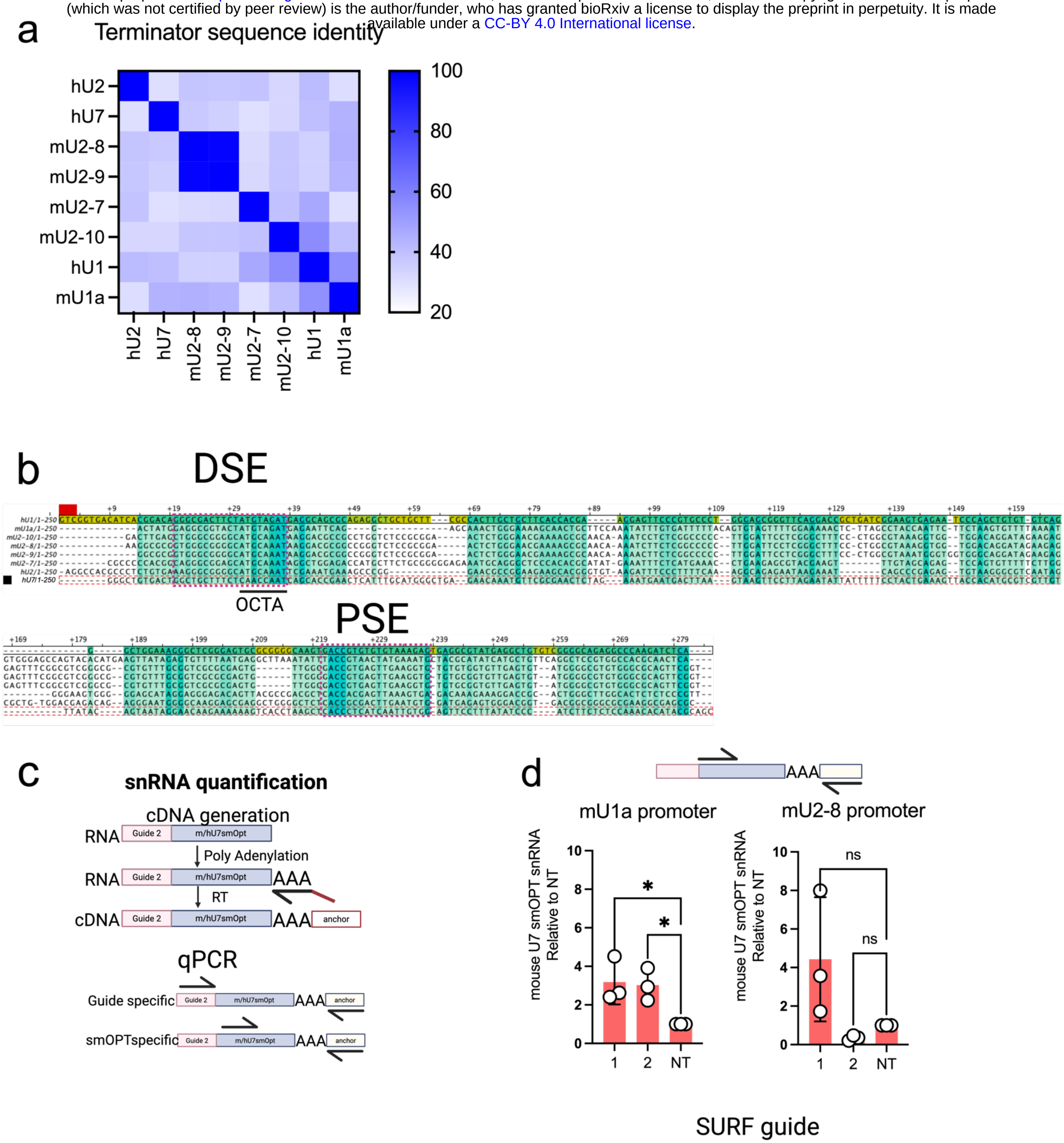

bioRxiv preprint doi: https://doi.org/10.64898/2025.12.01.691001; this version posted December 3, 2025. The copyright holder for this preprint (which was not certified by peer review) is the author/funder, who has granted bioRxiv a license to display the preprint in perpetuity. It is made available under a CC-BY 4.0 International license.

Figure S3: Extended data on novel promoters.

a) Heatmap showing percent nucleotide identity of the terminators used in this study.

b) Multiple sequence alignment of the promoters used in this study. Nucleotides are shaded by degree of conservation across sequences. The distal sequence element (DSE), proximal sequence element (PSE), and OCTA binding site are indicated.

c) Schematic of polyadenylation and reverse transcription used to generate cDNA for snRNA quantification for rt-qPCR.

d) Bar plots showing levels of snRNAs in iPSC-MN by rt-qPCR with forward primer against the snRNA backbone sequence, rather than the guide region used in Figure 2. Data are rt-qPCR of target RNA normalized to PuroR transcript levels (ΔCq), relative to normalized levels in cells transduced with non-targeting (NT) snRNA (ΔΔCq). One-way ANOVA with Dunnett's correction. N=3 biological replicates. *$P < 0.05$; ns, not significant.

bioRxiv preprint doi: https://doi.org/10.64898/2025.12.01.691001; this version posted December 3, 2025. The copyright holder for this preprint (which was not certified by peer review) is the author/funder, who has granted bioRxiv a license to display the preprint in perpetuity. It is made available under a CC-BY 4.0 International license.

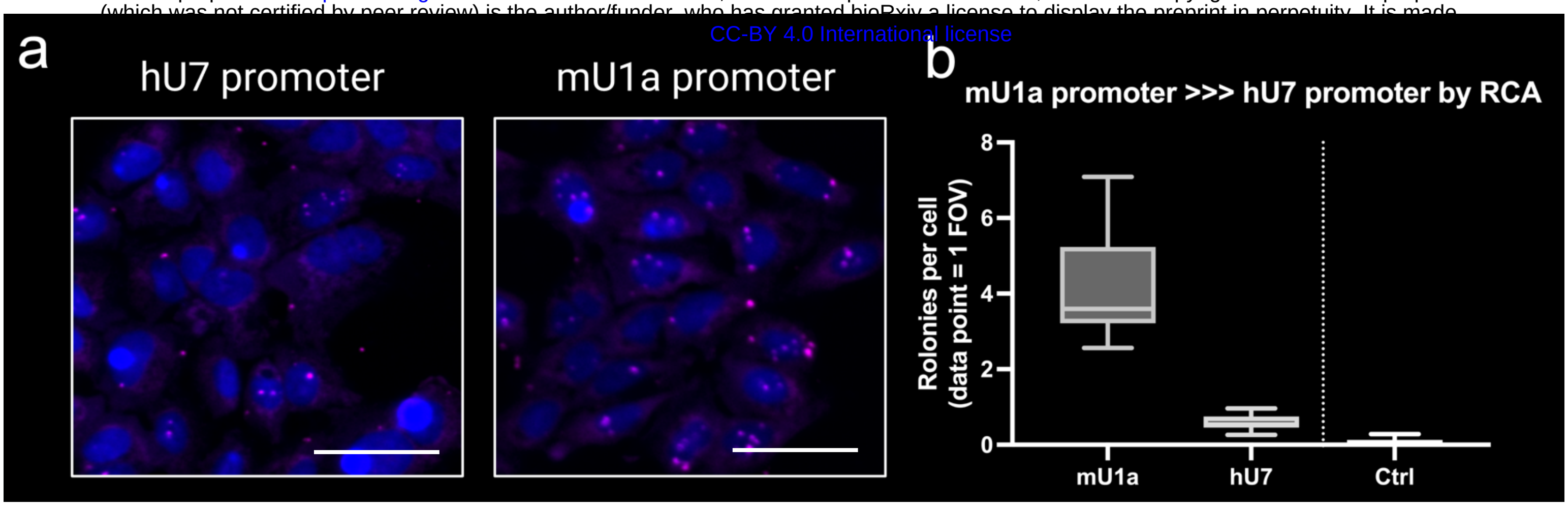

Figure S4: Rolling circle amplification detection of guide 2 sequence.
a) U2OS cells were transduced at low MOI with lentivirus expressing human U7smOPT with sg1 from the hU7 promoter or mouse U7smOPT with sg1 from the mU1a promoter. Scale bar = 40 µm.

b) Box plot showing the quantification of rolonies per field of view (FOV; N>7).

bioRxiv preprint doi: https://doi.org/10.64898/2025.12.01.691001; this version posted December 3, 2025. The copyright holder for this preprint (which was not certified by peer review) is the author/funder, who has granted bioRxiv a license to display the preprint in perpetuity. It is made available under a CC-BY 4.0 International license.

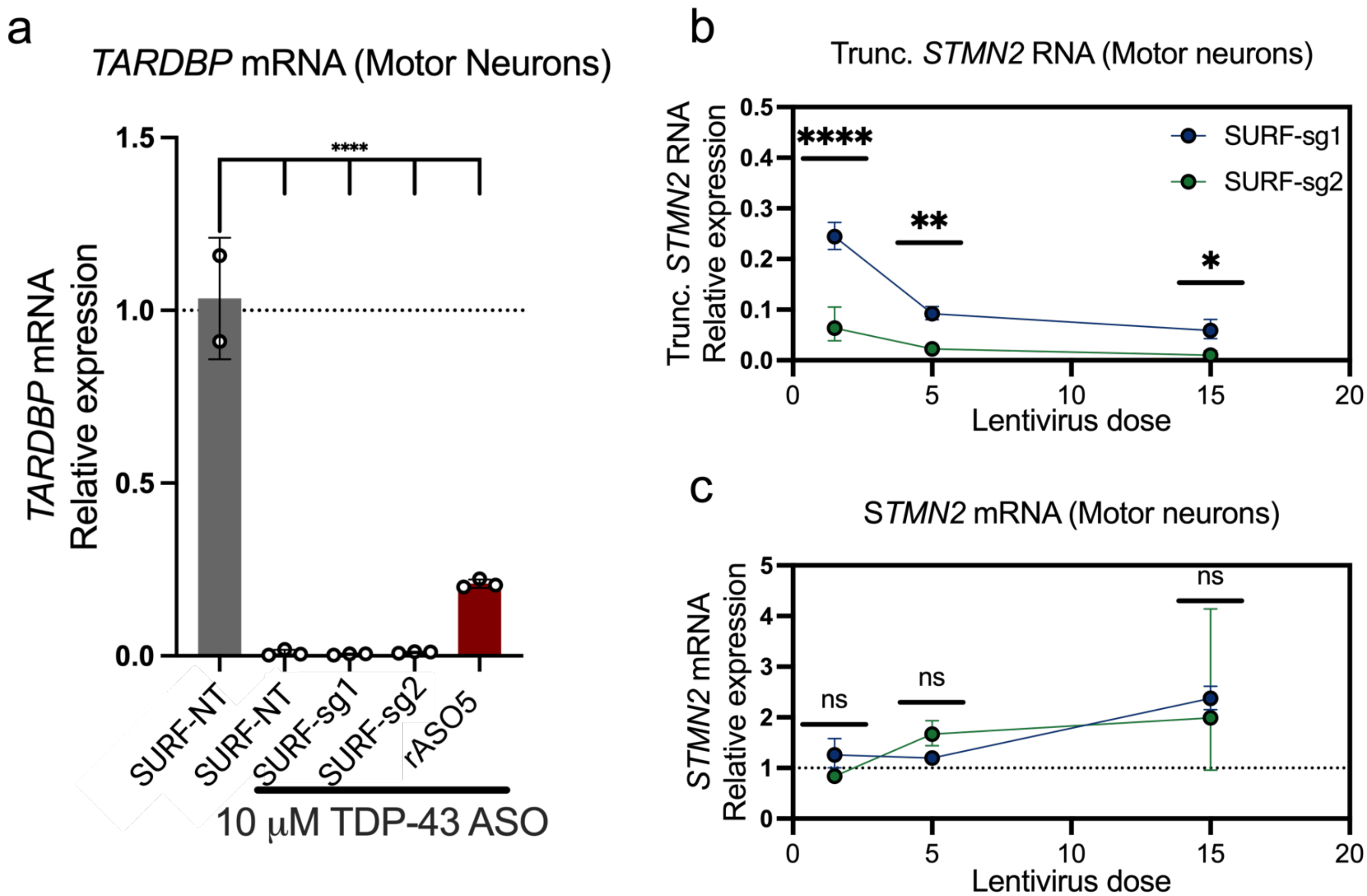

Figure S5: Extended data on suppression of cryptic splicing in iPSC derived motor neurons.

a) Bar plot of *TARDBP* transcript levels in iPSC-MN transduced with lentiviral vectors expressing the indicated snRNAs from a SURF cassette or treated with rASO5, following treatment of cells with either a *TARDBP* mRNA-targeting ASO or a non-targeting (NT) control. Data are rt-qPCR of *TARDBP* mRNA normalized to *GAPDH* mRNA, relative to normalized *TARDBP* mRNA levels in cells treated with NT ASO and transduced with SURF-NT expressing lentivirus. Lentiviral vector doses are the mid-level doses shown in Fig. 2f,g. N=2 biological replicates; SURF-NT; N=3 biological replicates; all other samples. One-way ANOVA with Dunnett's correction. ****P < 0.0001.

b,c) Line plot showing levels of truncated (b) and full-length (c) *STMN2* mRNA in iPSC-MN transduced with increasing relative doses of snRNA expressing lentivirus following ASO-mediated TDP-43 depletion. Data are rt-qPCR of target RNA normalized to GAPDH mRNA, relative to normalized levels in cells treated with (b) TDP-43 ASO or (c) control ASO and transduced with lentivirus expressing SURF-NT. N=3 biological replicates. One-way ANOVA with Dunnett's correction. ***P < 0.001; **P < 0.01; *P < 0.05; ns, not significant.

bioRxiv preprint doi: https://doi.org/10.64898/2025.12.01.691001; this version posted December 3, 2025. The copyright holder for this preprint (which was not certified by peer review) is the author/funder, who has granted bioRxiv a license to display the preprint in perpetuity. It is made available under a CC-BY 4.0 International license.

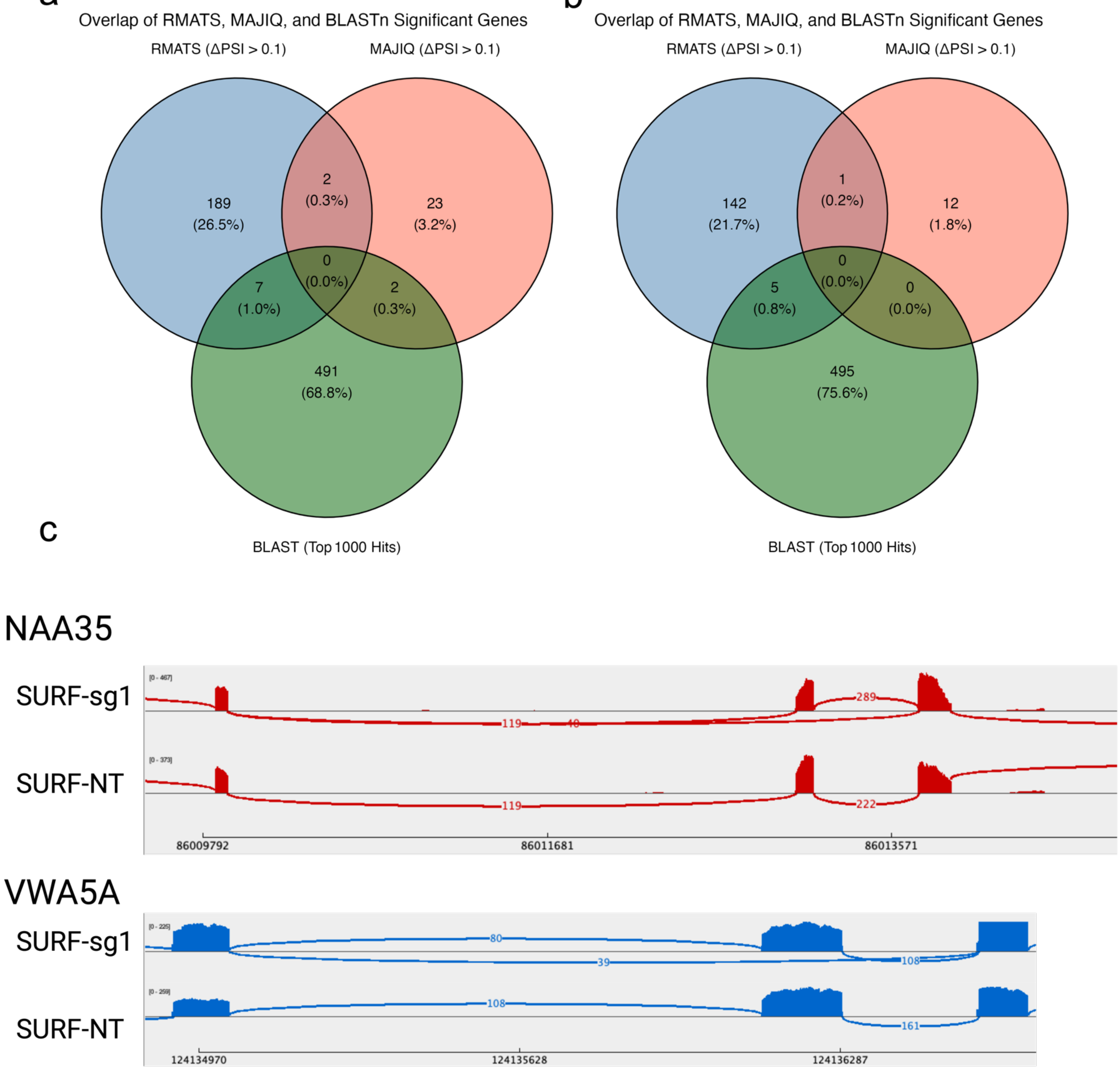

Figure S6: Characterization of off-target splicing events.

a,b) Venn diagrams of exon skipping events detected by rMATS, MAJIQ, and genes with regions of homology to the *STMN2* cryptic-exon targeting guide for SURF-sg1(left) and SURF-sg2 (right) compared to SURF-NT.

c) Genome browser views of skipped exons in *NAA35* (top) and *VWA5A* (bottom) identified for SURF-sg1 and SURF-NT.

bioRxiv preprint doi: https://doi.org/10.64898/2025.12.01.691001; this version posted December 3, 2025. The copyright holder for this preprint (which was not certified by peer review) is the author/funder, who has granted bioRxiv a license to display the preprint in perpetuity. It is made available under a CC-BY 4.0 International license.

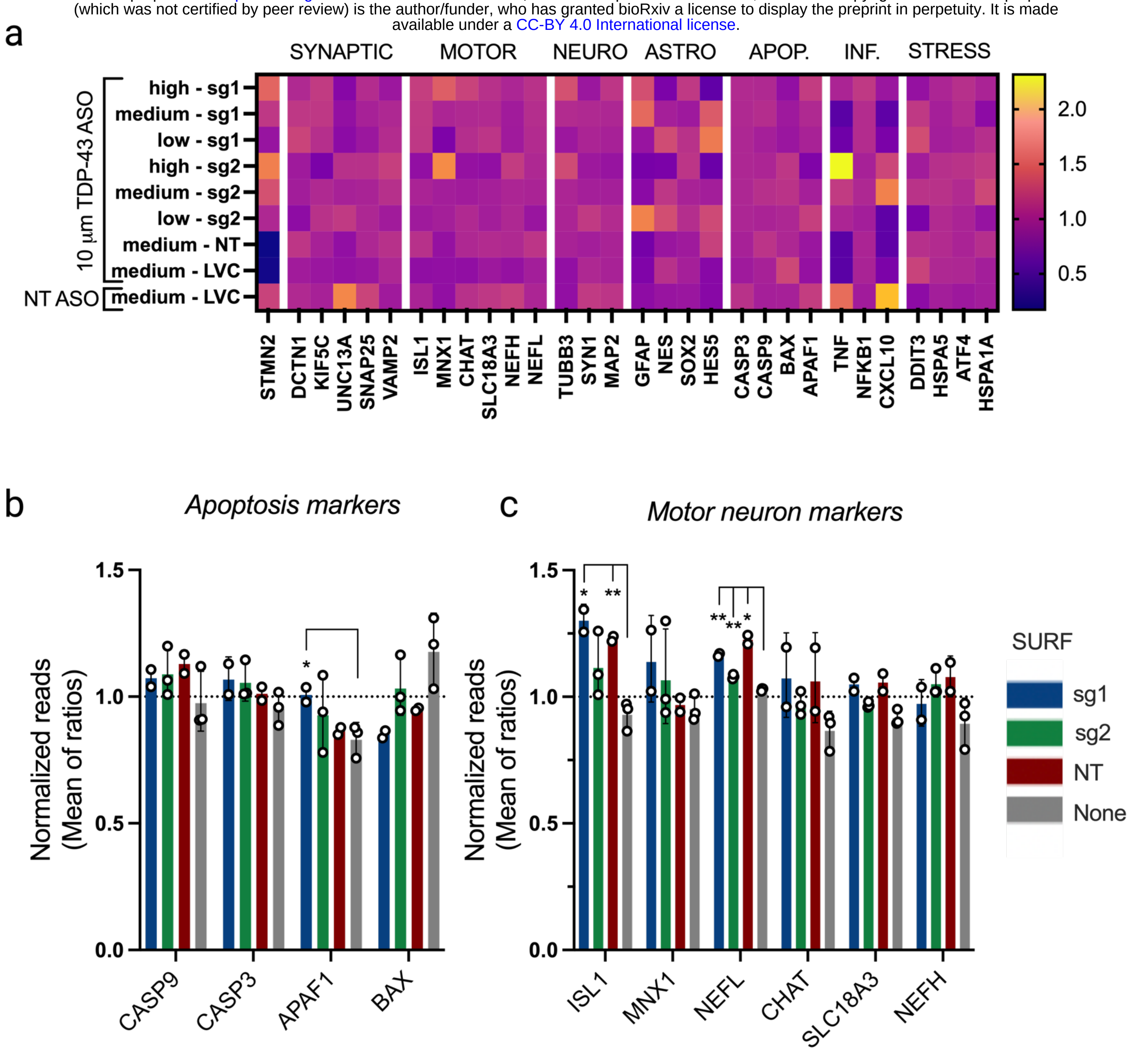

Figure S7: Expanded summary of RNA expression of genes relevant to neuronal health in iPSC-MN treated with SURF vectors.

a) Heatmap showing marker gene transcript levels in iPSC-MN transduced with either a high, medium, or low (15, 5, or 1 relative transduction units) dose of lentivirus expressing SURF-sg1, SURF-sg2 or SURF-NT with TDP-43 ASO. LVC indicates lentivirus control. Data are RNA-seq median of ratios methods (DESeq2) normalized to the average mean of normalized reads across all samples.

b,c) Levels of apoptosis marker (b) and motor neuron marker (c) transcripts in iPSC-MN transduced with sg1, sg2, or non-targeting (NT) sgRNAs, or in untransduced cells. Data are RNA-seq median of ratios methods (DESeq2) normalized to the average across all samples. N=3 biological replicates; sg2 and None; N=2 biological replicates; sg1 and NT. One-way ANOVA with Dunnett's correction. **P < 0.01; *P < 0.05; not significant unless otherwise indicated.

bioRxiv preprint doi: https://doi.org/10.64898/2025.12.01.691001; this version posted December 3, 2025. The copyright holder for this preprint (which was not certified by peer review) is the author/funder, who has granted bioRxiv a license to display the preprint in perpetuity. It is made available under a CC-BY 4.0 International license.

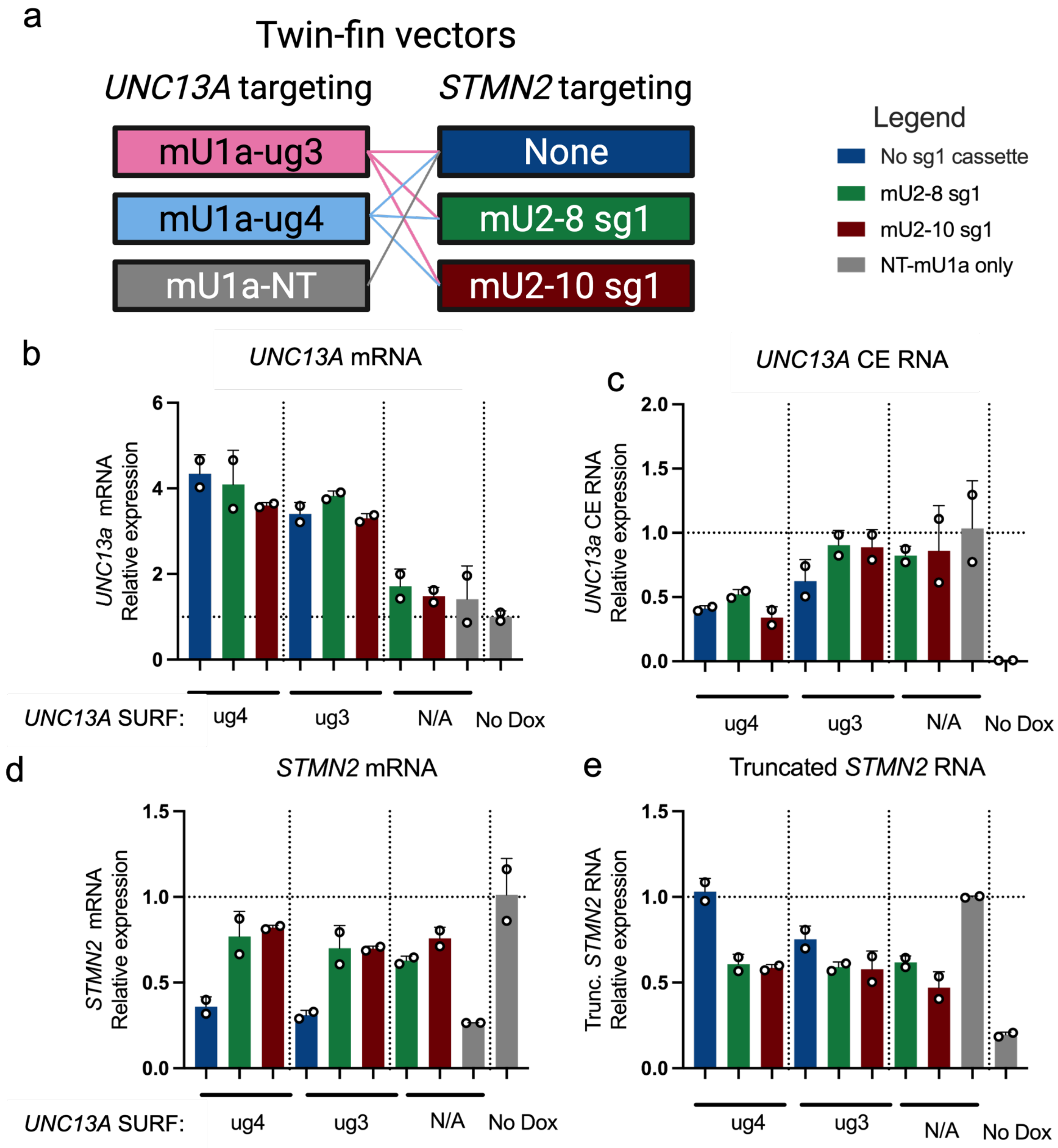

bioRxiv preprint doi: https://doi.org/10.64898/2025.12.01.691001; this version posted December 3, 2025. The copyright holder for this preprint (which was not certified by peer review) is the author/funder, who has granted bioRxiv a license to display the preprint in perpetuity. It is made available under a CC-BY 4.0 International license.

Figure S8: Results of screening ug3 and ug4 with sg1 in a single vector.

a) Diagram depicting the combinations of snRNA cassettes screened here, including ug3- and ug4-targeting designs under the mU1a promoter tested with sg1 expressed under mU2-8 or mU2-10.

b-e) Bar plots showing levels of (b) full length *UNC13A*, (c) CE-containing *UNC13A*, (d) full-length *STMN2*, and (e) truncated *STMN2* isoforms in a stable SH-SY5Y cell line expressing a shRNA targeting *TARDBP* from a doxycycline-inducible promoter and induced or undinduced cells (No Dox). Cells were treated with either a single snRNA vector or dual snRNA vector targeting one or both transcripts, or a non-targeting vector, as indicated. Data are rt-qPCR of target RNA normalized to GAPDH (ΔC_q), relative to normalized levels in cells transduced with and non-targeting snRNA ($\Delta\Delta C_q$). For full-length transcripts, $\Delta\Delta C_q$ was calculated using the uninduced control. For mis-spliced transcripts, $\Delta\Delta C_q$ was calculated relative to the doxycycline- treated control. The mean is shown by each bar plot. N=2 biological replicates.

bioRxiv preprint doi: https://doi.org/10.64898/2025.12.01.691001; this version posted December 3, 2025. The copyright holder for this preprint (which was not certified by peer review) is the author/funder, who has granted bioRxiv a license to display the preprint in perpetuity. It is made available under a CC-BY 4.0 International license.

Vector	Length	Reads	Percent of total
	4788	148	17.5980975
	3866	122	14.50653983
	2514	112	13.31747919
	6383	69	8.20451843
	2116	130	15.45778835
	1071	109	12.960761
	695	113	13.43638526
2xSURF(mU1a)	3425	38	4.51843044
	4837	150	32.32758621
	2490	98	21.12068966
	1854	133	28.6637931
TwinFin	3459	83	17.88793103

Table S1: AAV genome assemblies
